# Decolonising humanitarianism or humanitarian aid?

**DOI:** 10.1371/journal.pgph.0000179

**Published:** 2022-04-25

**Authors:** Tammam Aloudat, Themrise Khan

**Affiliations:** 1 Managing Director, Global Health Centre, Graduate Institute, Geneva, Switzerland; 2 Independent Development Practitioner and Researcher, Karachi, Pakistan; PLOS: Public Library of Science, UNITED STATES

Decolonising humanitarian aid has imposed itself on the humanitarian debate over the past few years, both in academic and practitioner circles. *Decolonisation*, as a term, alludes to the imbalance of power and the potential to rebalance this power asymmetry. But the how, to what extent, and with what means is not yet clear.

The use of decolonisation as a framework for action has thus far sidestepped the important—and more complex—act of understanding the monopoly, misuse, or abuse of power in the mainstream humanitarian sector. This lack of a collective consciousness has opened spaces for defensiveness, denial, and repudiation by those in the humanitarian sphere who see the call to decolonise as a threat to the power hierarchy. Likewise, decolonisation as an act is not seen equally by those who advocate it and ranges from reforming the system to calls for disposing of it all together.

Whether decolonising humanitarianism means giving back agency and leadership to the people it serves, combatting its embedded structural racism, or decentralizing its power and resources to local humanitarian actors, there is a need to talk of existing power imbalances across the Global North and South, if humanitarianism is to live up to its own claims.

## Humanitarianism vs. humanitarian aid

The conflation of humanitarianism and humanitarian aid is one of the features that allow a continuation, and often glorification, of a “humanitarian industrial complex” that is often complicit in the harm and violence that befalls people it seeks to help [1].

A clear distinction must be made between *humanitarianism*—the active belief in the equal value of all human life and the consequent action to assist others, protect their rights, and accept and promote their agency and worldview—and the current form of *humanitarian aid*—the formalized system of governments, agencies, and organizations largely based in and led by the Global North.

The lack of a distinction between the two, which is rarely acknowledged in the discussions on decolonisation, illustrates the pathologies of humanitarian aid, its historical entanglements with colonialism and politics, its engagement with power, and its complicity in extending disasters [2, 3]. For instance, Europe’s pushback against refugees and asylum seekers from outside Europe has led to countless deaths in the Mediterranean–according to the UN Agency for Migration, over 2000 migrants were recorded dead and missing in 2021 alone—as well as sending “illegals” back into the humanitarian disasters they are fleeing—without actually addressing the disasters themselves [4]. Thus, the European Commission, the world’s second largest humanitarian aid donor, added to the crisis instead of resolving it.

These issues must be examined from multiple angles including their explicit coloniality, displacement of politics, and structural racism and white supremacy [5–7]. A set of three cases close to the authors’ experiences can be used to illustrate some of these issues. It is important to mention here that while we point our critique squarely at the humanitarian aid regime, we do not do so at the individuals working in it, many of whom put their lives at risk, to provide aid within some of the most complex emergencies.

## Pakistan earthquake, 2005

The 2005 earthquake in northern Pakistan killed over 85,000 people and displaced almost three million. The response to this and other crises in Pakistan, including the floods of 2010 that displaced almost seven million people, included many Pakistanis who turned out in full force to donate, volunteer, and coordinate relief efforts, either independently, in collaboration with local NGOs, or with Government and Northern-led operations. However, the predominant narrative was not of how Pakistanis banded together to offer humanitarian assistance to their own, or how adept they were at responding to humanitarian disasters. Rather, the international humanitarian community chose to focus on its own response to the disaster, thus ignoring local efforts. It was the expatriate humanitarian workers from the Global North who spoke on behalf of the victims of disasters, distributed (branded) packages, and issued “communication” materials, ensuring that “their” contributions were front and center. Pakistani organizations such as the Edhi Foundation, on the other hand, the largest charitable philanthropic foundation in Pakistan, was never asked by global humanitarian organizations during the crisis: “What do you need from us?” Instead, they were and continue to be the invisible force who is first on the scene of any disaster, but who is never recognized for upholding humanitarianism over humanitarian aid.

## The Syrian civil war, 2011-present

As one of the world’s largest ongoing conflicts and displacements, the Syrian civil war has seen massive attempts at engagement by humanitarian actors of all sorts. But here too, there were several cases of Syrian-led organizations, saving lives on a daily basis [8]. A highly notable organization who provided search and rescue for victims of aerial bombardment across the country was the Syrian Civil Defense (White Helmets), which operates around 3000 volunteers across the country and which by 2017, rescued or aided more than 114,000 civilians from the rubble of their homes [9]. The White Helmets, a humanitarian actor by all accounts, except perhaps for their lack of a European headquarters, have instead, been targeted by a vicious information warfare to discredit their work [10, 11]. It might be argued that the positionality of the White Helmets justifies such responses as they do not claim neutrality and have had an explicit role in exposing the atrocities of the Syrian regime and its allies. This position may not be in line with humanitarian aid conventions, but clearly aligns with the ethos of humanitarianism. The White Helmets did receive some Northern funding, however, their operational choices remained local. But ultimately, the White Helmets were effectively abandoned by the international humanitarian community, reflecting the abandonment of a political or radical role of humanitarian aid and the system’s complicity with political oppression [12].

## COVID-19 and vaccine nationalism

As we write this paper, several changes are affecting the availability and distribution of vaccines for COVID-19 which still rages globally. Vaccine equity is still a gaping hole in crisis response as rich countries continue to deprive poorer countries of affordable access to vaccines, despite the growing threat and impact of variants. The pandemic demanded global action both morally and pragmatically, but instead, most of the countries of the Global North resorted to protectionism and narrow nationalistic interest. The reality saw HICs ordering vast amounts of vaccines, blocking patent waivers and knowledge sharing, and leading to a situation referred to as “vaccine nationalism or vaccine apartheid” [13]. This situation, described by WHO as a “catastrophic moral failure,” is an illustration of how with even destruction on a global scale, humanitarianism is not at the forefront. Even calls to help India’s humanitarian COVID catastrophe went unheard by the world’s biggest humanitarian agencies until the situation threatened to spill across borders. The “moral failure” of those who have not only come to represent the world’s biggest humanitarian aid donors, but also as they see it, the world’s biggest humanitarians, has exposed the gap between selfless doing good for humanity and doing good for oneself.

## Bridging the gap

Humanitarian aid, as it stands today, has been instrumentalized to serve the continuation of centuries of colonialism, warmongering, and economic exploitation; all of which continue in different shapes and forms. An example of this is the ongoing annihilation of the Palestinians. The attack on Gaza in May 2021 was one of the largest humanitarian disasters beyond the pandemic. As men, women, and children were ruthlessly killed, the humanitarian aid community stood largely silent or gave muted calls to “stop attacks’’ creating a false equivalence of guilt between the Palestinians in Gaza and the massive war machine of the Israeli apartheid regime. A community of governments and institutions, who are both morally and socially obliged not to turn a blind eye to suffering, but who do by picking and choosing which humanitarian causes to support, cannot continue to use the veneer of apolitical humanitarian action to be co-opted in the politics of global hegemony.

To ensure that humanitarian engagement moves from the position of willing complicity to active solidarity, we see the following as moral imperatives:

- Move away from a Eurocentric, White savior view of humanitarian interventions.
- View humanitarian functions as separate from the geopolitical hegemony of the Global North.
- Move away from the pretense of “apolitical” humanitarianism. The attempt of depoliticizing humanitarianism is in itself a political position that accepts the status quo and delegitimizes any challenge to the current world order.
- Link humanitarian aid with other social justice issues such as the action against racism, coloniality, and the effects of the climate crisis etc.
- Move away from making decisions on behalf of people to following their lead and providing technical assistance and resources when they need it.
- Prioritize Indigenous humanitarian actors in all countries who shoulder the burden of assistance. The international community should be working for them, not vice versa.
- *Editorial note*: *An earlier version of this piece was published on the PLOS blog*, *Speaking of Medicine and Health*, *on 13 July 2021*
